# *In planta* transformation methods to accelerate the domestication of perennial grain crops

**DOI:** 10.3389/fpls.2025.1638144

**Published:** 2025-07-17

**Authors:** Pedro M. P. Correia, Xinyi Dong, Mengming Chen, Anton Frisgaard Nørrevang, Guangbin Luo, Michael Palmgren

**Affiliations:** NovoCrops Center, Department of Plant and Environmental Sciences, University of Copenhagen, Frederiksberg, Denmark

**Keywords:** *In planta* transformation, recalcitrance, perennial agriculture, grain crops, monocots

## Abstract

The domestication of grasses has historically favored annual species due to their rapid growth and suitability for crop rotation; however, such crops rely heavily on human input. In contrast, perennial grasses, which live for multiple years, offer significant environmental benefits, such as improved soil health and natural resilience to biotic and abiotic stress, but have not yet been domesticated. Gene editing of yield-related genes presents an opportunity to improve yield stability in perennial cereal crops. However, this process typically requires transformation to introduce gene-editing tools, and many perennial grasses are recalcitrant to traditional *in vitro* transformation. Alternative *in planta* transformation methods have recently emerged, offering simpler, faster, and more genotype-independent approaches. These methods bypass the need for tissue culture and could potentially be used to transform recalcitrant plants more efficiently. In this review, we evaluate the potential of *in planta* transformation methods for developing perennial cereal crops and advocate for exploring the role of such crops in sustainable agriculture.

## Perennial grain crops as a sustainable alternative to annual cropping systems

Cereal grain crops, which are members of the grass family (Poaceae), include several widely cultivated species, such as wheat (*Triticum aestivum*), maize (*Zea mays*), rice (*Oryza sativa*), and barley (*Hordeum vulgare*), that provide the bulk of calories in the human diet (FAO, 2017). Annual grasses were domesticated over thousands of generations, resulting in the selection and retention of gene variants underlying desirable traits. Although the selected traits benefit agricultural production, artificial selection often works against natural selection, resulting in domesticated crops with reduced fitness, an inability to survive outside their cultivation areas, and a reliance on intensive chemical management (Chen et al., 2015; Meyer et al., 2012). Annual cropping systems involve replanting crops each year, which can negatively affect soil health. Frequent tillage contributes to soil degradation, resulting in erosion, greenhouse gas emissions, and decreased soil fertility (Crews et al., 2018; Olsson et al., 2024). The environmental effects of annual cropping underscore the urgent need for a shift toward more sustainable practices.

**Figure 1:**
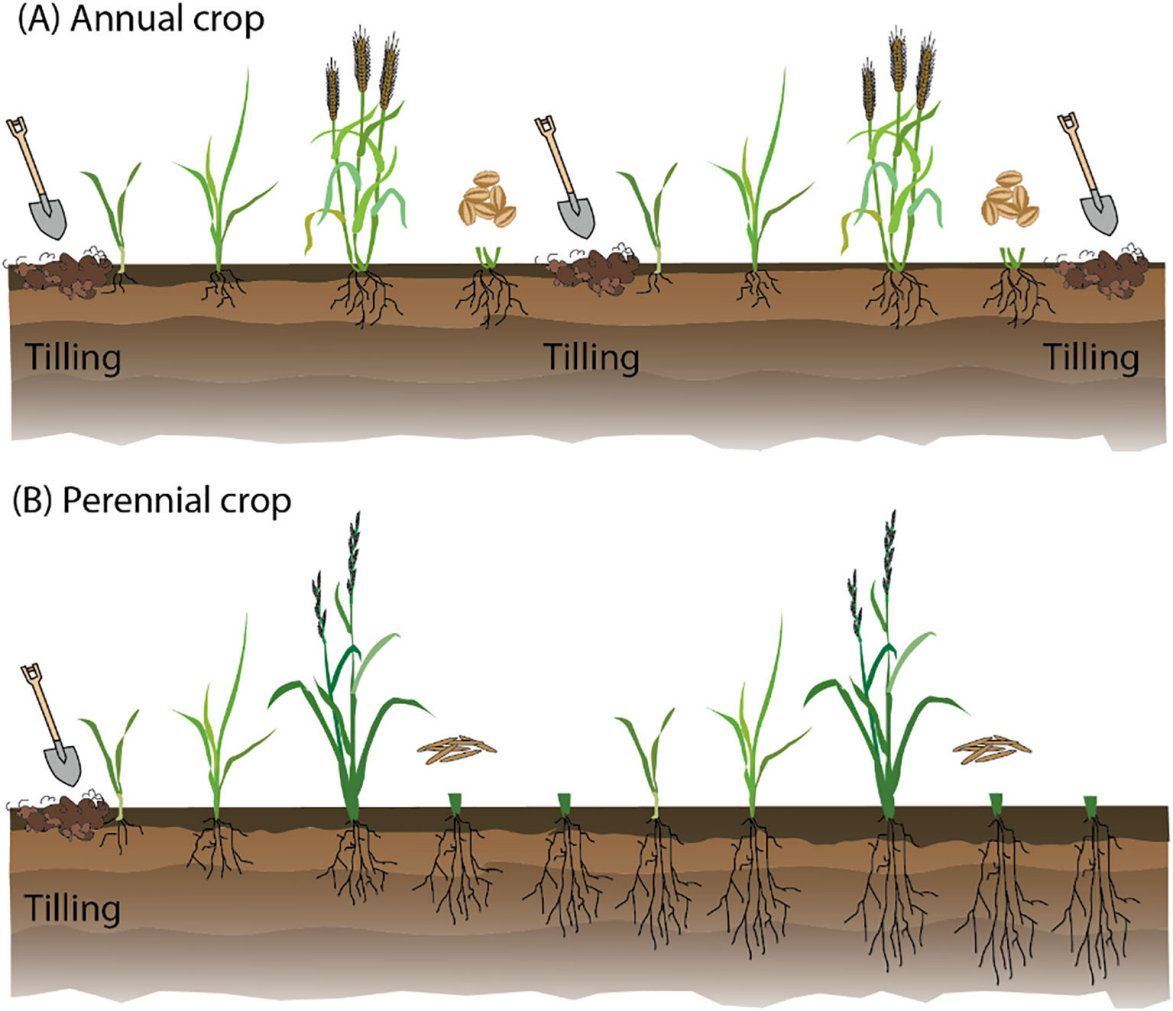
Comparison of annual and perennial cropping systems. **(A)** Annual crops live for a single season, requiring annual sowing and tilling, which promotes soil erosion, depletion of organic matter, and CO_2_ loss. These crops are highly dependent on human input for fertilization and protection against abiotic and biotic stress. **(B)** Perennial crops are sown only in the first year. These plants develop deep and extensive root systems that help preserve soil organic matter, reduce erosion, and improve overall soil health, and offer a sustainable alternative to annual crops.

Perennial agriculture offers numerous advantages over traditional annual cropping systems. Perennial crops have extensive root systems that reduce soil erosion and benefit soil health by improving soil structure and increasing organic matter content. Perennial crops also enhance water use efficiency deep in the soil, making the plants more resilient to drought and reducing the need for irrigation (**Figure 1**). Additionally, perennial systems sequester more carbon, contributing to the mitigation of climate change. Economically, these practices reduce the need for annual replanting, which lowers labor and input costs while offering more stable income streams for farmers (Chapman et al., 2022; Crews et al., 2018; Olsson et al., 2024; Paul et al., 2024). By improving soil health, water use efficiency, and biodiversity, perennial agriculture presents a sustainable alternative that could help address food security and environmental sustainability issues (**Figure 1**).

## Accelerating the domestication of perennial grain crops through genome editing

Efforts are underway to develop perennial cereal crops through wide hybridization—i.e., crossing existing annual crops with perennial relatives—and by traditional breeding of wild perennial species. The improvement of perennial intermediate wheatgrass (*Thinopyrum intermedium*) through traditional breeding (Bajgain et al., 2022) and the development of perennial rice PR23 from the hybridization of Asian cultivated rice (*Oryza sativa* ssp. *indica*) with its African wild perennial relative (*Oryza longistaminata*) (Zhang et al., 2022) are two successful examples of progress toward developing high-yielding perennial cereal crops.

However, high levels of ploidy and heterozygosity in perennial grasses mask alleles that are potentially useful for domestication, particularly in the case of recessive loss-of-function mutations (Østerberg et al., 2017), thereby complicating traditional breeding strategies. Targeted mutagenesis has emerged as a promising alternative tool for accelerating the domestication of new perennial crops. Leveraging an unprecedented understanding of crop domestication processes, the first step in these strategies involves precisely mutating a few genes using novel genome-editing technologies (Østerberg et al., 2017; Chapman et al., 2022; Luo et al., 2022).

There are over 7,000 perennial grass species that remain largely unexplored for domestication purposes (Frawley et al., 2020). Resources such as the Perennial Agriculture Project Global Inventory (PAPGI; http://www.tropicos.org/Project/PAPGI) provide information for assessing the potential utility of undomesticated perennial species (Ciotir et al., 2019). Allied with new targeted mutagenesis methods such as genome editing, these resources could prompt the cultivation of a range of novel perennial alternatives to annual crops. Such practices may become common if, for example, the EU lifts regulations on gene-editing tools such as clustered regularly interspaced palindromic repeats/CRISPR-associated protein9 (CRISPR/Cas9).

## Biological challenges in transforming perennial grasses

Genome editing has unlocked the ability to make precise and predictable changes to domestication genes, thereby paving the way for the accelerated domestication of wild perennial grain crops. Yet, the ability to transform and regenerate the target plant remains a prerequisite for successful genome editing. Although the technology for creating transgenic plants is decades old, the transformation of many plant species remains challenging, with high efficiency limited to a few species and even fewer cultivars within these species (Ahmar et al., 2023).

To date, genome-editing components have been delivered into annual cereal crops primarily through *Agrobacterium*-mediated or biolistic transformation of suitable explants, typically immature embryos. Numerous studies have explored strategies to enhance transformation efficiency and develop alternative tissue culture protocols (Ahmar et al., 2023). Several improved protocols have been published, mainly focusing on optimizing media compositions and refining procedures for *Agrobacterium*-mediated or biolistic transformation (Matres et al., 2021).

Many perennial grasses require exposure to cold (i.e., vernalization) or accumulated days of warmth for the vegetative to floral transition (Lundgren and Des Marais, 2020), which complicates access to reproductive tissues, such as immature embryos, for transformation. Another hurdle associated with the domestication of perennial grasses is their widespread self-incompatibility, which prevents self-fertilization and demands outcrossing (Baumann et al., 2000). Combined with high levels of ploidy and heterozygosity, this adds variability, complicating the optimization of tissue culture media compositions and transformation procedures. Self-incompatibility can also reduce seed set due to pollen abortion (DeHaan and Van Tassel, 2014), thereby affecting the availability of immature embryos as explants for transformation. Perennials often produce vegetatively propagated organs such as rhizomes and bulbous structures (Chapman et al., 2022); however, whether these organs can serve as substitutes for immature embryos in transformation has not been explored. These biological and technical constraints highlight the need for alternative strategies for transformation that bypass the reliance on immature embryos and tissue culture.

## *In planta* transformation

**Figure 2:**
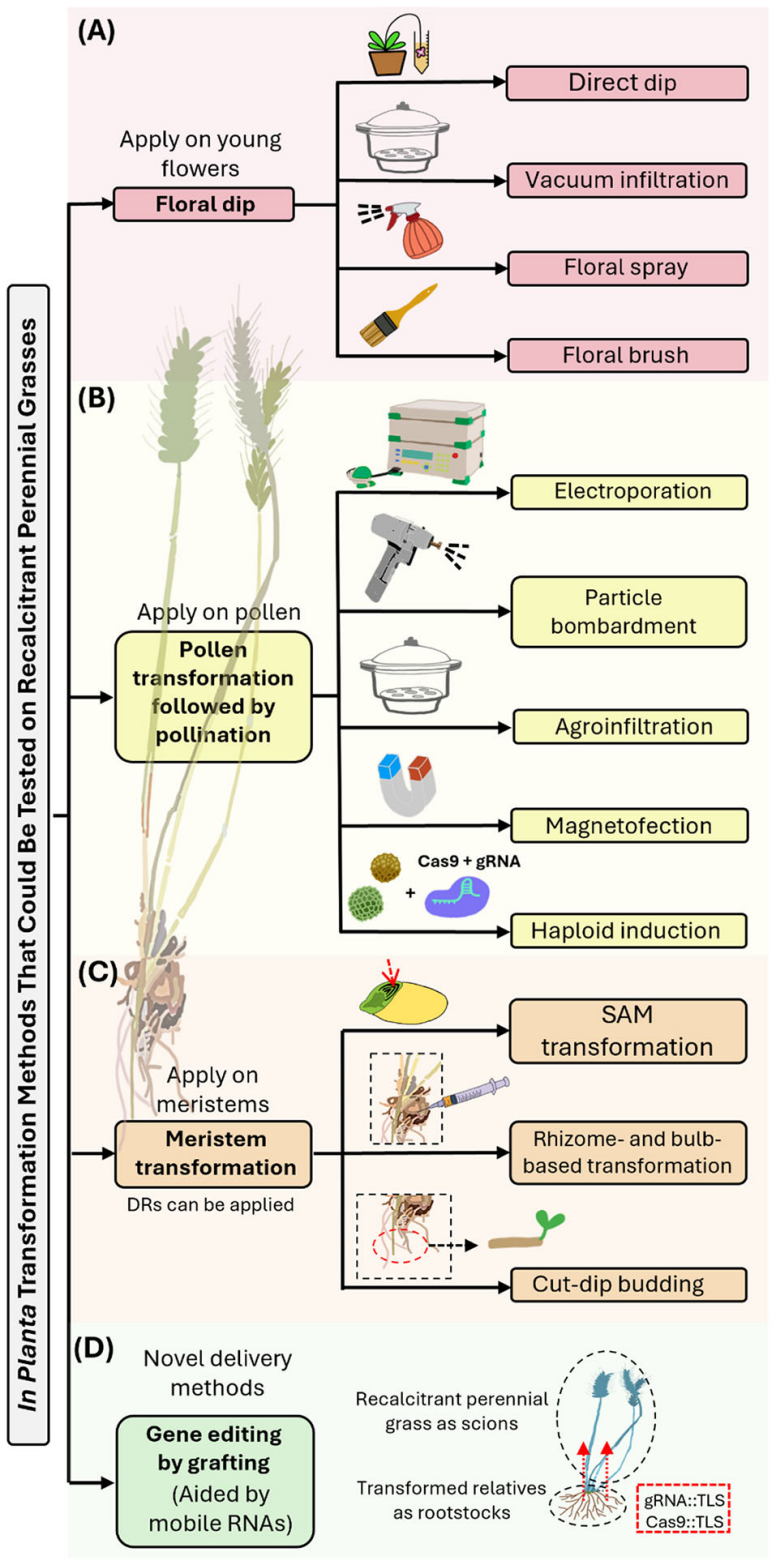
Overview of *in planta* transformation methods that could be applied to perennial grasses. **(A)** Floral dip transformation involves dipping young flowers in an *Agrobacterium tumefaciens* suspension, leading to transgenic seeds via natural fertilization. Methods include direct dip, vacuum infiltration, floral spray, and brush application. While these methods are effective in some annual grasses (Singh and Kumar, 2022), their efficiency in perennial grasses is limited by the unsynchronized anthesis and outcrossing nature of these plants. **(B)** Pollen transformation is used to deliver gene-editing tools into pollen grains, which are in turn used for pollination. Methods to overcome the challenges of pollen transformation include electroporation, particle bombardment, *Agrobacterium* infiltration, and magnetofection. Establishing efficient pollen transformation methods and using methods such as haploid induction editing (Kelliher et al., 2019) could facilitate the development of perennial grain crops. **(C)** Meristem transformation involves direct transformation into shoot meristems, which are composed of embryonic-type cells that divide to form new cells and organs. Methods include *Agrobacterium tumefaciens-*mediated transformation or bombardment to transform the exposed meristem tissues of embryos, seedlings, or mature plants. Targeting the cell layer that will develop into germ cells from the SAMs of mature embryos using CRISPR/Cas9 can bypass the need for tissue culture and be genotype-independent (Hamada et al., 2018, 2017; Liu et al., 2021; Luo et al., 2023; Tezuka et al., 2024), making it a potential method for perennial grain crops. Methods utilizing *Agrobacterium* to transform vegetatively propagated organs such as rhizomes and bulbous structures offer new avenues for the transformation of perennial grasses via these organs (Cao et al., 2023; Mei et al., 2024). Direct meristem induction by expressing developmental regulators (DRs) such as Wus2 and Bbm promotes embryo formation and can enhance transformation efficiency and regeneration speed (Lowe et al., 2018; Wang et al., 2023). **(D)** Novel delivery methods mediated by mobile RNAs, which carry genome-editing tools across plant tissues (Yang et al., 2023), could potentially be employed for targeted heritable gene editing in perennial grass crops.

*In planta* transformation, also known as *in situ* transformation, encompasses a diverse array of techniques aimed at directly integrating foreign DNA or editing a plant’s genome, followed by regenerating the cells into a whole plant. These approaches can utilize bacteria (e.g., *Agrobacterium* strains), viruses, or physical methods (e.g., particle bombardment) to deliver genetic material to cells (e.g., meristem tissues, reproductive tissues, embryos) and integrate it into their genomes (Bélanger et al., 2024). Transformed plants may consequently produce transformed seeds, enabling stable transformation or editing events in the next generation. Here we focus on *in planta* transformation approaches that have the potential to transform perennial grass species that are recalcitrant to *in vitro* transformation. We identify the bottlenecks of these methods and assess their potential for genome editing and domestication of perennial cereal crops (**Figure 2**, **Table 1**).

**Table 1:** Overview of *in planta* transformation in monocot grass species.

Species	Delivery Method	Plant Tissue	Expression	Target Gene	Plant Genotype	Efficiency (%)	Reference
Barley (*Hordeum vulgare*)	Virus-induced genome editing (VIGE)	Leaf tissues	CRISPR/Cas9	*CMF7, ASY1, MUS81, ZYP1*	Golden Promise (expressing Cas9)	T0: 17% - 35%	Tamilselvan-Nattar-Amutha et al., 2023
	Biolistics (iPB-RNP method)	Mature embryos	CRISPR/Cas9	*Ppd-H1*	Nishinohoshi	T0: 1% - 4.2% T1: 0.3% - 1.6%	Tezuka et al., 2024
Maize (*Zea mays*) Wheat (*Triticum aestivum*)	Haploid induction (HI-Edit)	Pollen/Egg	CRISPR/Cas9	*VLHP1, VLHP2, GW2, GT1*	Maize (GP721, GP650, ID5829, 412F) Wheat (AC Nanda, CMS)	T0: 0% - 8.8%	Kelliher et al., 2019
Maize (*Zea mays*), Sorghum (*Sorghum bicolor*)	Magnetofection	Pollen	Reporter gene	*GUS, GFP*	Maize (FFFMM, W22) Sorghum (Tx430)	Not effective	Vejlupkova et al., 2020
Perennial ryegrass (*Lolium perenne*)	Sonication-assisted *Agrobacterium-*mediated transformation (SAAT)	Seed, meristem tip	Overexpression	*IPT*	Grassland, Numan	T0: 14.2% - 46.65%	(Esmaeili et al., 2019)
Rice (*Oryza sativa*)	*Agrobacterium*-mediated transformation	Coleoptile	Overexpression	*GUS, DREB1A*	Phyongdo19	T0: 8.4%	Ho et al., 2023
		Mature embryos	Reporter gene	*GUS, GFP*	R207, Teqing	T0: 3.5% - 6.5%	Lin et al., 2009
		Mature embryos	Reporter gene	*GUS*	Koshihikari	T0: 40% - 43%	Supartana et al., 2005
		Seedlings	CRISPR/Cas9	*Expression of CAS9 and Hygromycin*	MR 219	T0: 9%	Tamizi et al., 2023
Sorghum (*Sorghum bicolor*)	*Agrobacterium*-mediated transformation	Seedlings	Reporter gene	*GUS*	SPV462	T0: 26% - 38%	Yellisetty et al., 2015
Switchgrass (*Panicum virgatum*)	*Agrobacterium*-mediated transformation	Seedlings	Reporter gene	*GUS*	Alamo	T0: 6% - 54% (transient expression)	(Chen et al., 2010)
Wheat (*Triticum aestivum*)	Biolistic DNA delivery	Mature embryos	Reporter gene	*GFP*	Fielder, Haruyokoi	T0: 1.39% - 2.28% T1: 0.7% - 0.87%	Hamada et al., 2017
	Biolistic DNA delivery	Mature embryos	CRISPR/Cas9	*GASR7*	Bobwhite	T0: 5.2% T1: 1.4%	(Hamada et al., 2018)
	Biolistics (iPB-RNP method)	Mature embryos	CRISPR/Cas9 RNP	*Qsd1, Or, HRGP*	Haruyokoi	T0: 1% - 8.3% T1: 0.9% - 2.1%	Kumagai et al., 2022
	*Agrobacterium*-mediated transformation	Mature embryos	Reporter gene	*GFP*	HD2894	T0: 22.5% T1: 3.33%	Kumar et al., 2024
	Biolistics (iPB method)	Mature embryos	CRISPR/Cas9	*Qsd1*	Haruyokoi, Yumechikara, Kitanokaori	T0: 0.5% - 2.51% T1: 0.5% - 1.68%	Liu et al., 2021
	Biolistics (iPB method)	Mature embryos	CRISPR/Cas9, GFP	*SD1*	Haruyokoi	T0: 0.17% - 0.86% T1: 0.08% - 0.34%	Luo et al., 2023
	*Agrobacterium*-mediated transformation	Flowers	Reporter gene	*GUS*	HD 2967, HD 3086, Bobwhite	T0: 0% - 0.23%	Singh and Kumar, 2022
	*Agrobacterium*-mediated transformation	Seedlings	Overexpression	*CP4-EPSPS*	HD2894	T0: 3.07%	Tarafdar et al., 2019

## Direct transformation into meristems

A major difference between annuals and perennials is that perennials have growing points (meristems) that remain indeterminate, allowing them to retain the ability to give rise to new tissues or organs after the first growing season. These indeterminate meristems are often located underground in bulbs, rhizomes, tubers, or corms, where they maintain vegetative growth and can undergo a developmental transition to generate a new plant.

*In planta* transformation of the indeterminate meristems of perennial grain crops may target the cell layer of the mature embryo that will develop into germ cells from the shoot apical meristem (SAM). CRISPR/Cas9 components, such as DNA vectors (Hamada et al., 2018, 2017; Liu et al., 2021, **Table 1**), ribonucleoprotein (RNP) (Kumagai et al., 2022, **Table 1**), and double-stranded DNA (dsDNA) donors (Luo et al., 2023, **Table 1**), can be coated onto gold particles for bombardment and delivery to a specific cell layer. This goal was first achieved in wheat (Hamada et al., 2017) and more recently in barley (Tezuka et al., 2024) (**Table 1**). However, the small size of seeds and their respective SAMs can make the delivery of CRISPR components challenging. Directly transforming embryonic cells with mobile Cas9 and single-guide RNAs (sgRNAs) capable of intercellular movement may increase the number of target stem cells in the meristem that undergo gene editing. This approach bypasses the need for tissue culture and is genotype-independent, but its potential for transforming perennial grasses remains to be tested (**Figure 2**).

Recent developments in nanoparticle-based delivery systems offer a promising alternative to particle bombardment (Wang et al., 2019). Nanomaterials such as carbon nanotubes, mesoporous silica nanoparticles, and lipid-based nanocarriers can also be explored for their ability to traverse the plant cell wall and deliver CRISPR/Cas9 components (Lowry et al., 2024; Wang et al., 2019). Key challenges include understanding how nanoparticle physicochemical properties, such as size, shape, surface charge, and aspect ratio can influence their ability to penetrate plant tissues and reach target cells (Hofmann et al., 2020). This approach bypasses the need for tissue culture and is genotype-independent, but its potential for transforming perennial grasses remains to be tested (**Figure 2**).

Mechanically injured embryos from mature seeds and SAMs from young seedlings have been used for transformation by imbibing the wounded plant material in an *Agrobacterium* solution. This technique has been used to transform rice (Supartana et al., 2005; Arockiasamy and Ignacimuthu, 2007; Lin et al., 2009; Ho et al., 2023; Tamizi et al., 2023; Sundararajan et al., 2023), sorghum (*Sorghum bicolor*) (Yellisetty et al., 2015), wheat (Kumar et al., 2024; Tarafdar et al., 2019), and perennial cultivars of ryegrass (*Lolium perenne*) (Esmaeili et al., 2019), and for transient transformation of perennial switchgrass (*Panicum virgatum*) (Chen et al., 2010) (**Table 1**). A modified approach for wheat uses embryos excised from mature seeds, which are centrifuged together with *Agrobacterium*, resulting in the direct transformation of the SAM (Ye et al., 2022). This method has been used for several plant species, but its potential for transforming perennial grasses has not been tested. One concern is that chimerism, caused by non-uniform transformation, can complicate the transmission of mutations to the progeny (Zlobin et al., 2020; Ye et al., 2022).

Recently, Mei et al. (2024) used the regenerative activity-dependent *in planta* injection delivery (RAPID) method to successfully transform several dicot species. They directly injected *A. tumefaciens* into the lower excised ends of stem segments of sweet potato (*Ipomoea batatas*) and bayhops (*Ipomoea pes-caprae*), as well as beneath the skin of potato (*Solanum tuberosum*) tubers. Transgenic progeny were obtained via regeneration and vegetative propagation at transformation frequencies of 12.5% to 40% (Mei et al., 2024). The RAPID system allows for direct gene transfer into regenerative plant tissues such as stem segments, tubers, rhizomes, and bulbs. This approach depends on the strong ability of transformed plants to undergo vegetative propagation, and further optimization of herbicide-based selection is needed to reduce chimerism (Mei et al., 2024; Zhong et al., 2024). The system has been used in sweet potato, potato, bayhops, and *Panax notoginseng*, providing a robust platform for tissue culture–free transformation. Since perennials often produce vegetatively propagated organs such as rhizomes and bulbous structures, they might also prove to be transformable using this strategy (**Figure 2**) However, this potential remains to be confirmed in monocot species, which differ significantly in their developmental and physiological responses.

Another method, the “cut-dip budding delivery system,” utilizes *Agrobacterium rhizogenes* to induce and transform hairy roots from the cut sites of explants (Cao et al., 2023). After generating transformed hairy roots with shoot-forming ability, transformed plants can be regenerated. The method involves infecting root segments by immersing them in an *A. rhizogenes* suspension (Cao et al., 2023). This technique has been successfully applied in a number of dicot species. Broader application to monocots might be facilitated by the use of disarmed *Agrobacterium* strains and by excision-based removal of integrated oncogenes (Cao et al., 2023). A similar transformation system remains to be tested in vegetatively propagated organs of perennial grasses (**Figure 2**).

## *De novo* induction of meristems

Meristem identity is, in part, dictated by developmental regulators (DRs). DRs work in conjunction with plant growth regulators, particularly the plant hormones cytokinin and auxin, to establish and maintain meristem identity. The expression of specific DRs in plant somatic cells can induce other developmental programs. In monocots, *Wuschel2* (*Wus2*) and *Baby Boom* (*Bbm*) promote somatic cells to form embryos that develop into whole plants (Lowe et al., 2018; Wang et al., 2023). By expressing *Bbm* and *Wus2*, transgenic monocot plants were successfully recovered using genotypes or explant types that were otherwise recalcitrant to genetic transformation (Wang et al., 2023). Ectopic expression of the maize *Bbm* and *Wus2* genes in rice, sugarcane (*Saccharum officinarum*), sorghum, and perennial switchgrass had a similar effect, suggesting a conserved function among monocot species (Lowe et al., 2016; Xu et al., 2022). Additional DRs, such as GROWTH-REGULATING FACTOR 4 (GRF4), GRF-INTERACTING FACTOR 1 (GIF1) chimera, and WUSCHEL RELATED HOMEOBOX 5 (WOX5) have shown potential for increasing transformation efficiency and the speed of regeneration (Debernardi et al., 2020; Wang et al., 2022; Yang et al., 2024). The use of DRs might expand the application of *Agrobacterium* infection of meristems to more plant species (Lian et al., 2022; Maher et al., 2020), including perennial grasses (**Figure 2**).

## Mobile RNAs as carriers of genome-editing tools

In plants, messenger RNAs (mRNAs) can move to neighboring cells via plasmodesmata and over long distances through the vascular system to regulate various biological processes in target organs (Kitagawa et al., 2024). The first evidence of RNA mobility emerged from studies on RNA viruses, whose movement proteins enable their intercellular movement from an infected cell to neighboring cells, allowing systemic virus spread (Deom et al., 1987; Wolf et al., 1989). Subsequent investigations revealed a more intricate mechanism that involves transfer RNA-like structures (TLSs) at the 3′-ends of RNAs (Zhang et al., 2009, 2016). Such TLSs are abundant in transcripts found in the phloem sap and serve as mobility signals. In transgenic Arabidopsis (*Arabidopsis thaliana*) lines, co-transcription of mRNA with TLSs triggers the systemic movement of mRNA between roots and shoots (Heeney and Frank, 2023; Kehr et al., 2022). Remarkably, the transported mRNA components are translated into functional proteins in the receiving cells (Zhang et al., 2016).

Building on this foundational work, the mobility of target RNA has been exploited for genome editing. For example, sgRNAs can be mobilized to the shoot apex, as demonstrated using viral vectors to transform transgenic Arabidopsis plants expressing Cas9. By fusing sgRNAs with mobile *Flowering Locus T* (*FT*) transcripts or TLS sequences, heritable gene editing was enhanced (Ellison et al., 2020). In a more recent study, Cas9 RNA and gRNAs were tagged with TLS motifs, allowing both types of RNA to be mobilized across graft junctions. The mobile transcripts efficiently moved from the transgenic rootstock to the wild-type scion, resulting in targeted heritable gene editing in the scion (Yang et al., 2023). The Grafting-Based Gene Editing approach offers a transgene-free alternative for genome editing by delivering mobile gene-editing signals across graft unions. While this technique has been successfully demonstrated in Arabidopsis and *Brassica rapa*, its broader application is currently constrained by graft incompatibility, particularly in monocot crops (Yang et al., 2023). However, the recent development of a micrografting method for monocots (Reeves et al., 2022) presents an opportunity to achieve transgene-free targeted gene editing in major staple crops and potentially in new perennial crops (**Figure 2**). To overcome transformation limitations in recalcitrant perennial species, this strategy could use model lines with well-established transformation protocols as rootstocks. For instance, transgenic cereal grain crops such as wheat, transformable wheat cultivar *Fielder* (Hayta et al., 2019), which have robust transformation systems could serve as donor rootstocks for delivering mobile CRISPR/Cas components to grafted scions. Recent research has shed light on the molecular basis of graft formation, particularly the genetic control of graft attachment and vascular reconnection (Feng et al., 2024; Notaguchi et al., 2008). These discoveries may lead to the emergence of new graftable combinations, and engineering transgenic rootstocks to enhance grafting compatibility could facilitate genetic editing through grafting junctions.

## Pollen transformation

Gene-edited plants have been obtained by delivering gene-editing tools into pollen grains (Bélanger et al., 2024; Toda et al., 2023). Here, the pollen grains are transformed and subsequently used to pollinate the recipient egg *in vivo*, resulting in nonchimeric transformation. Major challenges of this method include the thick cell wall of the pollen grain and the release of nuclease enzymes during pollen germination, as these factors hinder pollen grain transformation and the integration of exogenous DNA, respectively (Eapen, 2011). To overcome these obstacles, various methods could be utilized, including electroporation (Obermeyer and Weisenseel, 1995), particle bombardment (Touraev et al., 1997), *Agrobacterium* infiltration (Tjokrokusumo et al., 2000), and magnetofection, which uses magnetic force to enhance gene delivery (Zhao et al., 2017) (**Figure 2**). These techniques have been applied with varying degrees of success across different annual grass species such as maize and sorghum (Eapen, 2011; Toda et al., 2023), but with inconsistent results reported between different laboratories (Vejlupkova et al., 2020; Zhao et al., 2017; **Table 1**). Some grass species have mechanisms that enhance outcrossing by increasing the probability of pollen dispersal from one plant and its subsequent receipt by an unrelated plant (David and Pham, 1993). Establishing an efficient pollen transformation method for outcrossing perennial grasses could be advantageous (**Figure 2**).

A method involving haploid induction editing (Hi-Edit) technology has been used to edit elite lines of maize and wheat (Kelliher et al., 2019; **Table 1**). Here the paternal or maternal plant is a transformable cultivar that has been stably transformed with Cas9 and gRNA. The transformed plant is used as a pollen donor. However, the donor plant contains a mutation in *MATRILINEAL* (*MATL*) that eliminates the donor plant’s genome. After fertilization, the Cas9 and gRNA are expressed from the donor plant’s sperm or egg cell, editing the recipient’s chromosome and resulting in the elimination of the donor plant’s chromosome. This results in editing without the need for transformation and the production of a transgene-free edited elite crop (Kelliher et al., 2019). Using this approach, already established transformation systems could be used to edit a recalcitrant species such as a perennial grass (**Figure 2**). Hi-Edit has been used to deliver genome-editing components into maize and Arabidopsis, reducing chimerism and simplifying the production of edited lines. While efficient, improvements in haploid-doubling protocols are required to enhance their applicability (Kelliher et al., 2019).

## Virus-mediated transformation

RNA viruses infect plants and move systemically within the plant. Tobacco rattle virus (TRV), potato virus X (PVX), and barley stripe mosaic virus (BSMV) have been used as vectors to introduce RNA sequences into plants without the need for transformation or regeneration (Awan et al., 2023; Tamilselvan-Nattar-Amutha et al., 2023). However, because Cas9 exceeds the cargo capacity of most viruses, it is usually only feasible to deliver sgRNAs, which requires that the plant host is already a transgenic Cas9-expressing line (Ellison et al., 2020; Liu et al., 2024, 2022, 2023). Endonucleases that are smaller and more compact may serve as alternatives to Cas9. In a recent study, a transposase-associated TnpB endonuclease was packaged with a guide RNA in the RNA virus TRV. Following infection of Arabidopsis with the engineered virus, editing was achieved in a single step (Weiss et al., 2025). This finding suggests the potential of using virus-induced genome editing to transform recalcitrant perennial grasses.

## Genotype-independent fast transformation

Zhong et al. (2024) introduced Genotype-independent Fast Transformation (GiFT) as a rapid *in planta Agrobacterium*-mediated transformation system for soybean (*Glycine max*). The GiFT method uses wounded germinated seeds as explants, followed by a brief liquid culture phase under sublethal herbicide selection and direct soil transplantation, during which continued *in planta* selection ensures the preferential regeneration of transgenic shoots. This approach enabled the recovery of healthy, non-chimeric T_0_ plants within approximately 35 days without extensive tissue culture. GiFT demonstrated high transformation frequencies across a diverse range of elite and recalcitrant soybean varieties and was further validated for CRISPR-Cas12a-mediated genome editing applications. The general applicability of this novel *in planta* transformation method remains to be tested.

## Conclusion

Perennial grasses are typically recalcitrant to transformation using traditional *in vitro* methods. More direct *in planta* transformation protocols have recently been shown to be successful for the transformation of several dicots and annual grasses, but whether these methods can be applied to perennial grasses remains to be determined. We hope that this review will inspire researchers focused on developing sustainable agricultural practices to test whether *in planta* transformation methods can be used to overcome the transformation and genome-editing bottlenecks associated with the improvement of perennial grain crops.
